# Polystyrene-colonizing bacteria are enriched for long-chain alkane degradation pathways

**DOI:** 10.1371/journal.pone.0292137

**Published:** 2023-10-03

**Authors:** Shu Wei Hsueh, You-Hua Jian, Sebastian D. Fugmann, Shu Yuan Yang

**Affiliations:** 1 Department and Institute of Biomedical Sciences, College of Medicine, Chang Gung University, Kweishan, Taoyuan, Taiwan; 2 Institute of Biomedical Sciences, College of Medicine, Chang Gung University, Kweishan, Taoyuan, Taiwan; 3 Department of Nephrology, Linkou Chang Gung Memorial Hospital, Kweishan, Taoyuan, Taiwan; 4 Department of Obstetrics and Gynecology, Linkou Chang Gung Memorial Hospital, Kweishan, Taoyuan, Taiwan; Universidade Catolica Portuguesa, PORTUGAL

## Abstract

One of the most promising strategies for the management of plastic waste is microbial biodegradation, but efficient degraders for many types of plastics are still lacking, including those for polystyrene (PS). Genomics has emerged as a powerful tool for mining environmental microbes that may have the ability to degrade different types of plastics. In this study, we use 16S rRNA sequencing to analyze the microbiomes for multiple PS samples collected from sites with different vegetation in Taiwan to reveal potential common properties between species that exhibit growth advantages on PS surfaces. Phylum enrichment analysis identified Cyanobacteria and Deinococcus-Thermus as being the most over-represented groups on PS, and both phyla include species known to reside in extreme environments and could encode unique enzymes that grant them properties suitable for colonization on PS surfaces. Investigation of functional enrichment using reference genomes of PS-enriched species highlighted carbon metabolic pathways, especially those related to hydrocarbon degradation. This is corroborated by the finding that genes encoding long-chain alkane hydroxylases such as AlmA are more prevalent in the genomes of PS-associated bacteria. Our analyses illustrate how plastic in the environment support the colonization by different microbes compared to surrounding soil. In addition, our results point to the possibility that alkane hydroxylases could confer growth advantages of microbes on PS.

## Introduction

It is widely accepted that the plastic waste accumulated and continuously increasing in the world presents one of the gravest environmental challenges we currently face. One potential solution is to mine the diverse world of microbes that carry a broad range of enzymes for the ability to degrade different types of plastics. The most efficient examples known to act on plastics are those that degrade polyethylene terephthalate (PET) into monomers, such as the PETase-MHETase enzyme combination isolated from *Ideonella sakaiensis* and leaf branch compost cutinase [1, 2].

For the biodegradation of polystyrene (PS), several studies have reported modest results by individual microbial strains and consortia [3–9]. In 2015, larvae of yellow mealworms (*Tenebrio molitor*) were shown to ingest and degrade the extruded form of PS (EPS, or Styrofoam) [10]. Since then, the larvae of several other species including dark mealworm (*Tenebrio obscurus*), superworm (*Zophobas atratus*), wax moth (*Galleria mellonella*), and beetles have also been shown to exhibit similar behavior and abilities [11–14]. Mineralization of PS in *Tenebrio molitor* occurs in the gut via the help of the associated microbiota [11, 15], but few enzymes and pathways that mediate such degradation have been defined.

PS, like polyethylene (PE) and polypropylene (PP), has a carbon backbone that makes them highly refractory to biodegradation. In considering possible biological pathways that may lead to degradation of such plastics, an interesting point of reference is the degradation of aliphatic hydrocarbons which are similarly hydrophobic and contain a large portion of alkanes. Microbes are well-documented to perform bioremediation of oil-polluted environments by degradation of hydrocarbons [16]. These processes are frequently mediated by oxidation via alkane hydroxylases [17], and the similarities between the properties of petroleum hydrocarbons and plastics make alkane hydroxylases interesting candidates for degrading plastics whose polymer backbone consists of carbon-carbon bonds. Indeed, there is accumulating evidence indicating that alkane hydroxylases can transform PE [18], and these enzymes have also been implicated in oxidizing PS [11, 19, 20]. Of interest are AlkB and CYP153, two well-studied alkane hydroxylases that act on molecules with less than 20 carbons. In addition, there are hydroxylases that can act on longer chain alkanes such as AlmA and LadA, two proteins that have been reported to assimilate ones in the range of C_20_-C_36_ [21, 22]. These enzymes are intriguing as plastic molecules have a carbon backbone and are very long polymers.

To investigate the potential of bacterial enzymes for PS degradation, we surveyed the microbiomes of multiple EPS waste samples from various environmental sites by 16S rRNA sequencing. Functional analyses revealed that hydrocarbon-metabolizing microorganisms are enriched in PS-associated species, and subsequent examination of the presence of alkane hydroxylases revealed that microbes possessing genes involved in long-chain alkane degradation show the strongest enrichment. These observations highlight long-chain alkane hydroxylases as novel candidates of PS biodegradation.

## Methods

### Sample collection and 16S rRNA gene sequencing

EPS waste pieces were collected at 7 different sites of varying location, vegetation, and pH, and two control soil samples were also collected from each site, one directly underneath the EPS sample (“S” for soil) and another 0.5 m away (“SN” for soil nearby). The pH values for all soil samples were measured with pH paper of soil mixed with equal parts of water (HYDRION acid-base test paper, Micro Essential Laboratory).

To extract environmental DNA from the EPS samples, the collected pieces were first submerged in no carbon media (26.1 mM Na_2_HPO_4_, 22 mM KH_2_PO_4_, 8.6 mM NaCl, 18.7 mM NH_4_Cl, 0.4 mM MgSO_4_, 36 mM FeSO_4_, 29.6 mM MnSO_4_, 47 mM ZnCl_2_, 4.6 mM CaCl_2_, 1.3 mM CoSO_4_, 1.4 mM CuSO_4_, 0.1 mM H_3_BO_3_, 34.2 mM EDTA, 1.8 mM HCl), vortexed for 5–10 min, and subjected to DNA extraction with the DNeasy PowerWater Sterivex kit (Qiagen) as instructed by the manual. DNA from soil samples was extracted with the DNeasy PowerSoil Pro kit (Qiagen) following manufacturer’s protocol. These DNA samples were submitted for full length 16S rRNA gene sequencing (forward primer: 5’ AGRGTTYGATYMTGGCTCAG; reverse primer: 5’ RGYTACCTTGTTACGACTT) on the PacBio SMRT platform and the average raw HiFi reads obtained per sample was 16055.

### Data analysis

The reads obtained for individual sequences were processed with DADA2 to obtain amplicon sequence variants (ASVs) which are then referenced with databases (NCBI, GreenGenes, SILVA, eHOMD, UNITE) to generate ASV tables that included taxonomy assignments. The ASV tables were then used for the determination of Simpson diversity indices and rarefaction curves to indicate alpha diversity, and for PCA analysis to calculate beta diversity. Functional enrichment analysis was performed with the FAPROTAX database which is built from published metabolic and functional data and used for comparing collective metabolic profiles between microbial samples [23].

To determine top enriched species associated with PS, the read numbers for each ASV was first normalized against total reads in a sample before calculating average enrichment values by dividing the average normalized reads of an ASV across PS samples with the average for either the S or SN samples (S3 Table). The equation we used for calculating the enrichment ratios is: log (10[(ASV_PS_/Sum_PS_)x100]/10[(ASV_C_/Sum_C_)x100]) in which ASV_PS_ and ASV_C_ represent the number of reads for a certain ASV in a given PS sample and its corresponding soil control S or SN samples; Sum_PS_ and Sum_C_ refer to the total reads in the same PS and control S or SN samples.

These calculations enabled the ranking by enrichment values of all species detected whose whole genome sequences are available to generate the top enriched species list. Control lists of species were selected randomly excluding species whose whole genomes were yet unavailable or that overlapped with the top 50 enriched list (S3 Table).

### Identification of alkane hydroxylases homologs

To identify homologs of alkane hydroxylases, well-characterized homologs of each type were used as the query sequence to blast against whole genome sequences (tblastn) of designated species and strains at the NCBI database and further examined for the presence of signature domains or residues if such information is available.

For AlkB, homologs were identified based on homology to AlkB from *Pseudomonas putida* (Q9WWW6) and the presence of conserved motifs including 3 histidine boxes (HELXHK, EHXXGHH, LQRHSDHHA) and an HYG motif (NYXEHYGL) [24]; those that belong to the LLM class of flavin-dependent oxidoreductase were excluded. The query sequences for identifying AlkG and AlkT homologs were also taken from *P*. *putida* (Q9WWW4 and Q9L4M8, respectively). The query sequence for identifying CYP153 homologs was from *Mycobacterium marinum* (B2HGN5).

For AlmA, the homolog from *Acinetobacter baylyi* (Q6F7T9) was used as the query. Hits that contained at least a region of 250 a.a. of 25–50% identity to the query and a predicted flavin-binding monooxygenase (FMO-like) domain or CzcO domain, both related to alkane hydroxylases, were called AlmA homologs. LadA homologs were determined similarly using the homolog from *Geobacillus thermodenitrificans* (A4IU28) as the query for tblastn, and the hits we called LadA showed at least 200 a.a. of homology to the query with 20–50% sequence identity. They also contained flavin-utilizing monooxygenase domains.

For methane monooxygenases, protein sequences from *Thauera butanivorans* (A7MAQ9), *Nocardioides sp*. *CF8* (E9L6F6), *Mycobacterium sp*. *TY-6* (Q08KF2), and *Pseudonocardia sp*. *TY-7* (Q08KE2) were used as the query for BmoR, BmoA, PrmA and Prm1A, respectively.

Classification of AlmA and LadA proteins included homologs identified in the top 30 PS-enriched species along with those from the random 60 species list as we wanted to include a greater number of homologs from species that did not exhibit enrichment on PS (S4 Table). Phylogenetic analyses of these homologs were carried out first by aligning the regions homologous to the AlmA and LadA queries with the ClustalW tool. Trees were subsequently built with MEGA7 using the Maximum Likelihood method and performing 1000 bootstrap replicates [25]; bootstrap consensus trees are presented.

### Comparisons of multiple *almA* genes in strains of a species

There is one fully assembled genome each available for *Pseudomonas toyotomiensis* (SM2) and *Pseudomonas alcaliphila* (JAB1), and 4 and 2 additional whole genome projects for the two species, respectively (*P*. *toyotomiensis*: KF710, JCM 15604, DSM 26169, 718, SM2; *P*. *alcaliphila*: JCM 10630, NBRC 102411). For all strains, homologs of AlmA were determined by tblastn as described above and in the Results section. In addition, the two genes immediately upstream and downstream to each *almA* were identified for synteny determination.

## Results

### Profiling of PS-colonizing microbiota

**Fig 1:**
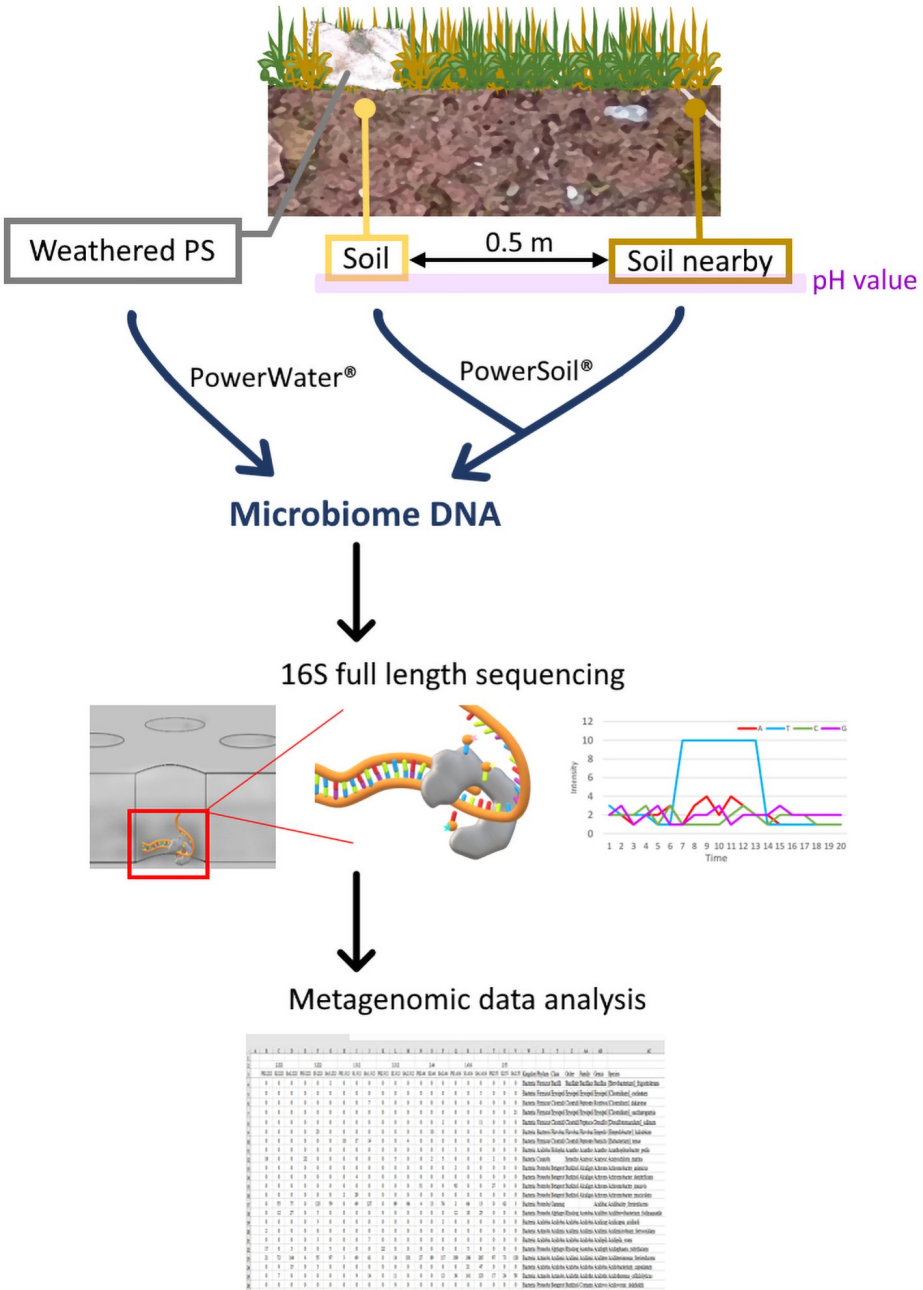
Diagram of sample collection and processing. Weathered EPS samples were collected from 7 different locations; at each location two control soil samples were also collected, one directly underneath the EPS piece (S for soil) and another 50 cm away (SN for soil nearby). DNA was extracted from the samples using commercial kits and subsequently submitted for full length 16S sequencing to survey the associated microbiomes.

To investigate the microbiomes on PS samples in natural environments, we collected 7 pieces of waste EPS from different sites in Taiwan (Fig 1). These samples were all partially embedded in surrounding soil and showed clear signs of weathering, suggesting that these EPS pieces have been in those environments for substantial amounts of time which could allow for microbial communities to colonize and mature. If any microbes have the potential to take advantage of the plastic material, such as using it as a survival niche or an alternative carbon source, those strains would gain growth advantages and exhibit enrichment compared to microbiota in the soil in their proximity. Due to these considerations, for each EPS waste piece we also collected two control soil samples, one directly underneath the EPS piece we collected (“S” for soil), and another one 0.5 m away (“SN” for soil nearby). For each site of collection, we recorded the vegetation around where the EPS sample was collected, and the pH values of the soil samples were determined (S1 Table). To analyze the bacterial microbiomes associated with the EPS and soil samples we collected, we performed 16S rRNA gene sequencing to determine the species composition and abundance for all 21 samples. We chose to sequence full-length 16S genes to achieve greater species resolution.

### PS-residing microbiota is significantly different from those in surrounding soil samples

**Fig 2:**
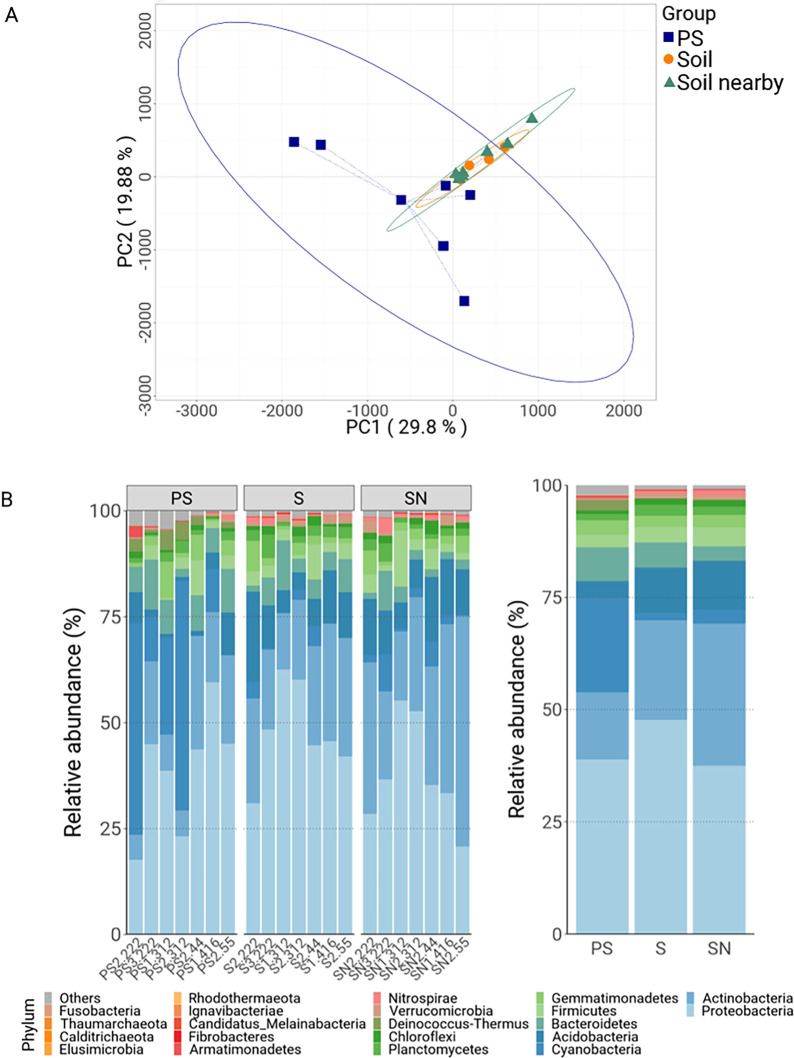
Comparisons of PS- and soil-associated microbiomes. **A**, PCA analysis of the microbiomes collected off PS (blue), S (orange), and SN (green) samples. **B**, Bar graphs showing the relative levels of the most-abundant phyla for the PS, S, and SN samples with individual specimens of each type displayed separately (left graphs) or collectively (right graph). Color codes of the different phyla are to the bottom of the graph.

The first test we applied to our genomics data was to investigate whether there are differences in the microbiota observed on PS samples compared to the soil ones. Using PCA, we found that bacterial contents on the PS samples as a group were significantly different compared to those of either the S or SN samples (Fig 2A). The microbial compositions on soil samples were quite similar to one another in spite of the differences in location, vegetation, and soil pH of the collection sites (S1 Table); in comparison there is much more variation in the microbiomes associated with PS samples. Another finding of note is that the alpha diversities of PS-associated microbiomes were less compared to those in soil (S1A, S1B Fig). This indicates that the PS surface supports colonization of a more limited repertoire of microbes, which is what some reports studying microbiomes on other petroleum-based plastics have also found [26, 27]. The differences between the three types of samples can also be observed when we examined the bacteria composition based on taxonomy. The abundance levels of the most prevalent phyla and species are different on PS compared to the soil controls (Fig 2B, S2 Fig), and the most highly enriched species on PS, S, and SN samples are also distinct as evidenced by analysis using the LEfSe method (S3 Fig) [28].

These analysis results, taken together, indicate that the microbial community colonizing EPS is significantly different from those found in the surrounding soil environments. This is in spite of the proximity of PS samples to the soil samples and that the microbes on PS most likely having originated from their immediate environments. We think this shows that PS, due to its chemical composition or acting as a niche, is able to nurture specialized localization and growth of microbes.

### Enrichment of bacterial phylum on PS samples

To investigate how PS-associated microbiomes are distinct from those of the surrounding environments, we examined whether there are certain bacterial clades that are particularly abundant in the PS samples compared to the soil controls. At the phylum level, we found that the abundances of Cyanobacteria and Deinococcus-Thermus on PS samples are clearly higher than those in controls (Fig 2B). Interestingly, the enrichment is due to multiple species from the two phyla being present and not due to the enrichment of one or two outstanding species (S2 Table), suggesting that common properties of species within these two phyla underlie their ability to survive on PS. One of the best known characteristics of cyanobacteria is their ability to perform photosynthesis, and we wondered whether the prevalence of cyanobacteria on the PS samples we collected was due to them being readily exposed to sunlight. However, the SN samples we collected were also surface samples that had ample exposure to sunlight, and the PS samples showed comparable enrichment in cyanobacteria compared to both the S and SN samples. Therefore, the higher abundance of cyanobacteria on PS samples is not due to the availability of sunlight.

The more interesting idea of why cyanobacteria exhibit PS enrichment would be that they are able to benefit from PS as a source of nutrients either by directly degrading PS or utilization of PS metabolites generated by other microbes. We did not find any metabolic pathways or enzymes of note in Cyanobacteria or Deinococccus-Thermus species enriched on PS compared to controls (S4A Fig), but this certainly does not rule out the possibility that other novel enzymes would be able to contribute to survival advantages of these phyla on PS.

### Genomics analysis reveal an enrichment of hydrocarbon degradation pathways in microbiota on PS samples

**Fig 3:**
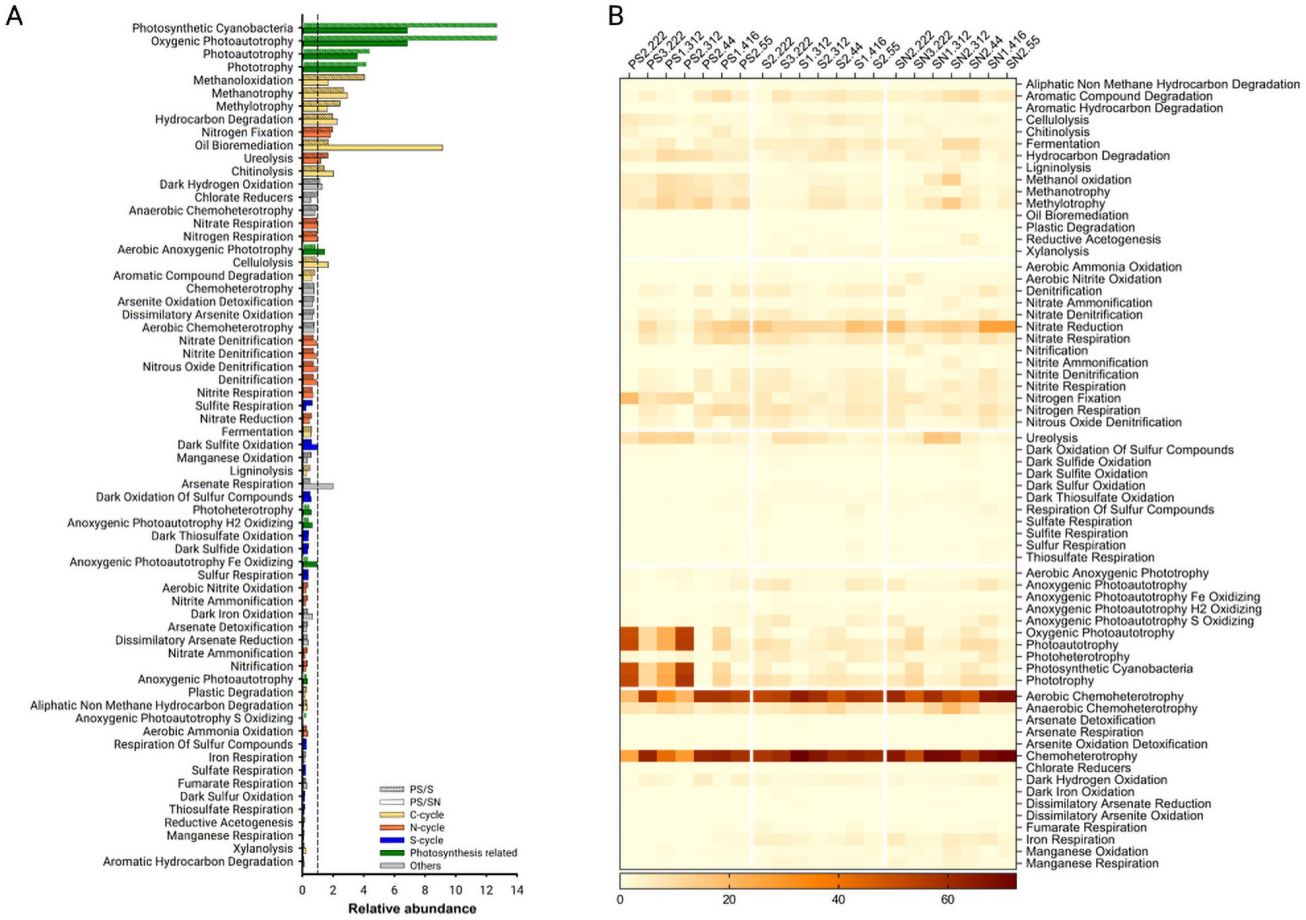
Analysis of relative abundances of metabolic pathways in PS- vs. soil-associated microbiomes. **A**, Differences in the abundances of various metabolic pathways between microbes on PS and soil samples are calculated by taking the ratios of average abundances in PS over S (solid bars) or PS over SN (hashed bars). Pathways are color-coded based on whether they are related to metabolism of carbon (yellow), nitrogen (orange), sulfur (blue), photosynthesis (green), or others (grey). **B**, Heatmap indicating the calculated abundances of each type of metabolic pathway in individual samples.

To investigate whether the bacterial community present on PS show signs of being able to utilize the plastic as a source of nutrients, we looked into whether there are metabolic pathways that are overrepresented in PS-associated species. Using the FAPROTAX database [23], we tested whether any metabolic pathway is more abundantly represented in microbes present on PS, and found strongest enrichment for two categories of pathways: photosynthetic functions and carbon metabolism, especially those related to alkane metabolism (Fig 3A). These enrichment effects are observed across the majority of PS samples, suggesting that the phenomena are common and robust (Fig 3B). The pathways related to photosynthesis included terms such as “Photosynthetic cyanobacteria” and “Photoautotrophy” which are not surprising as there is a clear enrichment of cyanobacteria among PS-associated microbes compared to soil control samples (Fig 2B).

The second type of enriched pathways are those related to alkane metabolism such as “Oil bioremediation”, “Methanotrophy”, “Hydrocarbon degradation”, among others (Fig 3A). The enrichment of these categories is not related to cyanobacteria as similar enrichment levels were observed when cyanobacteria species were removed from our sequencing results (S4B Fig). A significant proportion of petroleum hydrocarbons are alkanes with carbon lengths of 10–100, and there is an extensive literature on bacterial strains that can degrade different chain lengths of alkanes and petroleum hydrocarbons. PS also has a carbon backbone. Thus, the enrichment of alkane metabolic pathways raises the intriguing possibility that such enzymes are involved in PS colonization or biodegradation.

### Species enriched on PS frequently encode long-chain alkane hydroxylases

**Fig 4:**
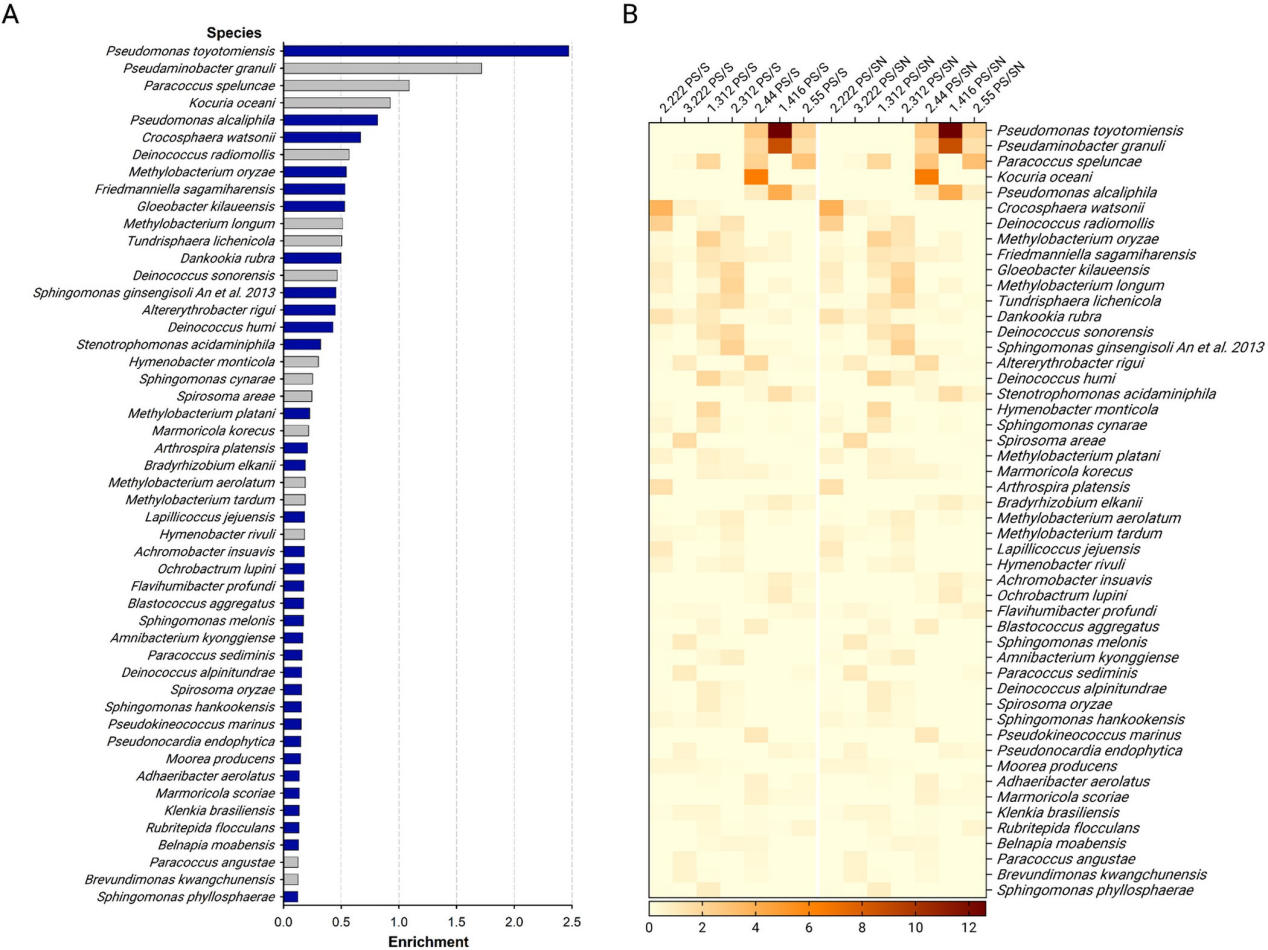
Species enrichment on PS compared to soil samples. **A**, Fold enrichment of the average presence of individual species on PS compared to the two soil samples. The bars for the species whose whole genome sequence is available are blue, those for species without are gray. **B**, Heatmap representation of individual species abundances comparing PS to S and PS to SN.

The enrichment in metabolic pathways present in PS-associated species related to alkane degradation prompted us to investigate whether enzymes involved in these processes are present in species found on PS. We first sorted the species profiled in our sequencing survey based on their PS-to-soil enrichment levels (Fig 4A, S3 Table). We calculated the average enrichment across all 7 sets of samples to favor those that exhibit repeat enrichment among collection sites (Fig 4B).

**Fig 5:**
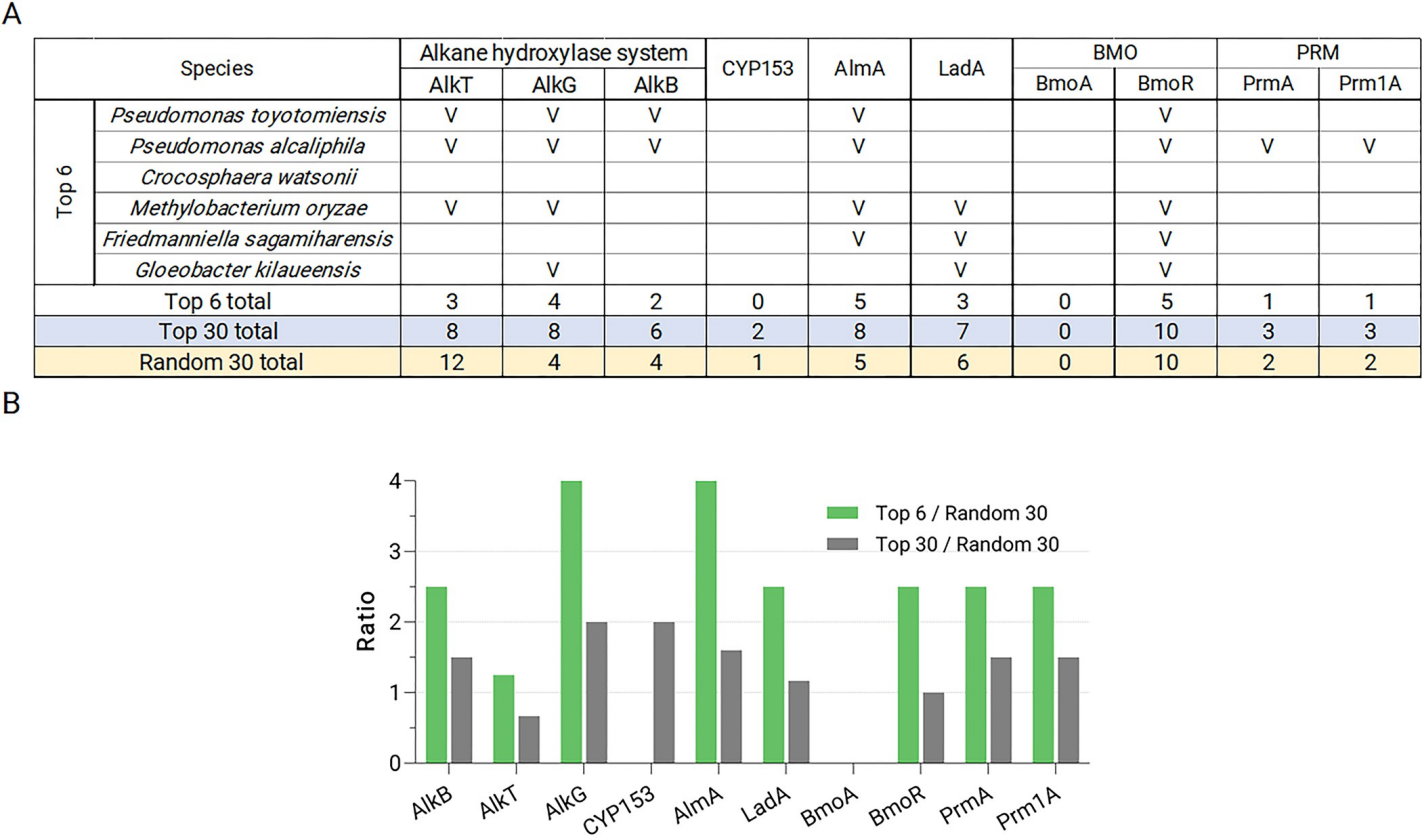
Presence of alkane hydroxylases in the NCBI reference genomes of top PS-enriched species and randomly chosen control species. **A**, The numbers of alkane hydroxylases found in the genomes of top PS-enriched species and random species. The numbers for the top 6 species, which are those that exhibit more than 50% PS-to-soil enrichment, are listed by individual species. **B**, Fold enrichment of the presence of alkane hydroxylases in top 6 and top 30 PS-enriched species compared to the 30 random species.

At the species level, few exhibited high fold-enrichment levels, but the enrichment of top candidates was clear (Fig 4A). The species with the highest average enrichment, *Pseudomononas toyotomiensis*, is one that was first isolated from soil immersed in hydrocarbon-containing hot springs and has been reported to assimilate hydrocarbons [29]. This further encouraged us to look in the genomes of the top enriched species for the presence of alkane hydroxylase homologs, and we targeted *alkB*, *CYP153*, *almA*, and *ladA* as they encode the main enzymes known to degrade hydrocarbons of various chain-lengths [17]. For AlkB and CYP153, we found that there is modest enrichment in the top 30 most PS-enriched species compared to 30 random species profiled in our sequencing data (Fig 5A and 5B, S4 Table). The top and random 30 lists included only those whose whole genome sequences are available as this is a prerequisite for homolog searches. Of note, the top two species on the list, *P*. *toyotomiensis* and *Pseudomonas alcaliphila*, both carry *alkB* genes in their genomes (Fig 5A). We further examined enrichment for AlkG and AlkT, two components integral to the actions of AlkB, and found that they also showed enrichment in the top 30 PS-enriched species compared to the random list. This result strengthens the possibility that the alkane hydroxylase system is overrepresented in the PS-associated species.

There is also mild enrichment for three of the four methane monooxygenases inspected (PrmA, Prm1A, BmoR), but the clearest enrichment observed was for long-chain alkane hydroxylases, AlmA and LadA (Fig 5B). Of the 6 species that exhibit at least 50% enrichment on PS over soil samples, 5 encoded at least one of AlmA or LadA. The prevalence of AlmA in top 30 species compared to the random 30 list is also the highest of all alkane hydroxylases we examined (Fig 5B). For the two most PS-enriched species, *P*. *toyotomiensis* and *P*. *alcaliphila*, we identified 3 AlmA homologs each, and for all strains of these two species whose genome sequences are available, the same 3 AlmAs are present (S5 Fig). Moreover, there is a high degree of synteny of the loci surrounding these *almA* genes; these loci are syntenic between different strains as well as between *P*. *toyotomiensis* and *P*. *alcaliphila* (S5 Fig). These results indicate that the *almA* genes are common and conserved in these two closely-related bacteria, thus while we have not directly profiled the genomes of these two Pseudomonas species, it is not unreasonable to consider the possibility that the strains we detected indeed encode AlmAs.

As in the examples of *P*. *toyotomiensis* and *P*. *alcaliphila*, many bacterial species encode more than one long-chain alkane hydroxylase in their genomes, and some carry both *almA* and *ladA* genes whereas others encode more than one AlmA or LadA proteins. Based on sequence comparisons, there appear to be multiple families of AlmA and LadA proteins [30]. To investigate whether some subtypes may be more strongly associated with colonization on PS, we compared the sequences of AlmA and LadA homologs identified in the top and random species. This was done by alignment of homologs and subsequent construction of phylogenetic trees to highlight structural similarities that could delineate functional categories.

**Fig 6:**
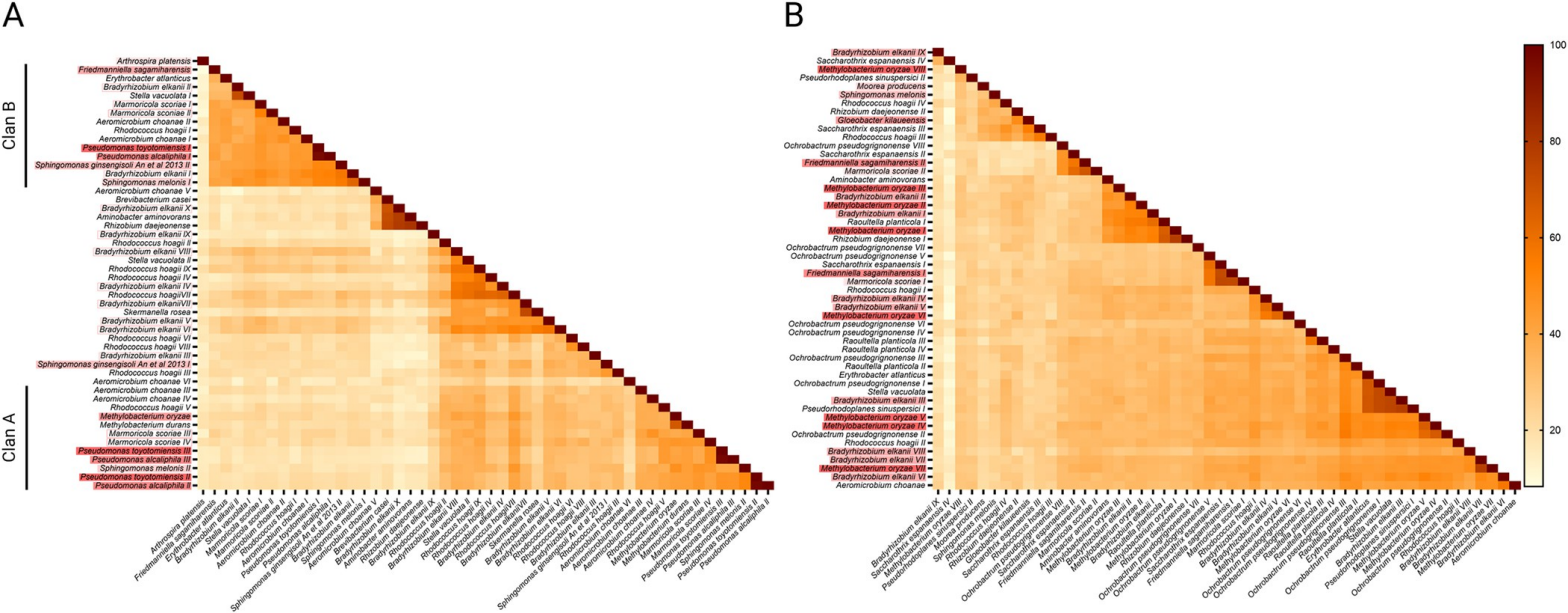
Pairwise comparisons of homologs of the two main long-chain alkane hydroxylase families, AlmA (**A**) and LadA (**B**). Percent identities between pairs are indicated as the color code on the right. Different homologs from a single species are designated with roman numerals, and the shading color of species name from dark red to none indicate strong to no enrichment of a given species on PS. In (A), two groups contain a high fraction of homologs from PS-enriched species, and they are assigned as Clans A and B.

Our phylogenetic analyses revealed considerable diversity within homologs of AlmA and LadA, and there are several major subtypes for both long-chain alkane hydroxylases (Fig 6A and 6B). We found that while LadA homologs from PS-enriched and random species were relatively evenly distributed across different subtypes (Fig 6B), there are two groups of AlmA—Clans A and B—that are more highly represented in top 30 PS-enriched species with Clan B containing the densest cluster of AlmA homologs from PS-enriched species (Fig 6A). Of the 5 top species that exhibit over 50% PS-to-soil enrichment and encode AlmA proteins, 4 had homologs in Clan B while only 1 out of 8 AlmA-encoding, non-enriched species had homologs placed in the same group. Furthermore, AlmA proteins from Clan B are also the ones with the highest similarity to the *Acinetobacter baylyi* AlmA. *Acinetobacter baylyi* is a species isolated from oil-polluted sites, and its AlmA is one of the best-characterized long-chain alkane hydroxylases [31]. These again connect the biodegradation of petroleum hydrocarbons to abilities in PS colonization.

## Discussion

Biodegradation of plastics is a great vision, but its realization still faces many significant challenges. An important starting point for the development of such technology is to identify microbial species and enzymes that have activity in plastics biodegradation so that we can start to understand and mine the potential in the microbial world for treating the plastic waste problem we face.

In this study, we profiled 7 sets of PS-associated microbiome along with control soil samples near and around the site of PS collection to investigate the relationship between such plastic waste and environmental microbes. These analyses have given rise to two main insights: that select phyla are highly enriched on PS waste, and that enzymes involved in alkane hydroxylation are possible contributors to bacterial colonization on PS surfaces.

The vegetation and soil pH of our collection sites were different, and these would presumably affect the microbiome in their environment, but our sequencing analysis revealed that the major microbes present in the soil of different collection sites were rather similar. In contrast, the 16S rRNA profiles of the 7 PS samples we obtained were much more different. The PS-enriched bacteria are not absent in the soil samples but just at much lower frequencies, suggesting that select microbes in the natural environments can gain survival advantages on the PS surface due to differences in nutrient utilization or other properties conferred by the plastic.

### Alkane degradation pathways are enriched in PS-associated microbiota

For the PS-associated microbiota, functional analysis of metabolic pathways using reference genomes from the NCBI database revealed enrichment in two physiological categories: photosynthesis and alkane metabolism. The former is due to cyanobacteria being enriched on PS samples, and the latter pointed to the possibility that bacteria that encode alkane degradative enzymes are selected to grow on PS surfaces. It is very tempting to propose that certain alkane hydroxylases are reactive towards PS, thereby conferring survival advantages of microbes that produce these enzymes and are able to colonize on PS surfaces. However, we need to point out that the data we present here does not establish such a direct relationship.

Currently, the best-studied alkane hydroxylases are AlkB and CYP153 that process alkane with carbon chain lengths up to about 20. The presence of CYP153 in the genomes of PS-enriched species was sparse whereas AlkB is encoded by the two top species. This is a mild enrichment and could be functionally related to PS colonization especially considering that there are recent studies that reported AlkB-encoding species with the ability to biodegrade PE which also has a carbon backbone [11].

Plastics are very long polymers, thus we further examined the genomes of PS-enriched strains for the presence of long-chain alkane hydroxylases AlmA and LadA as these two are the major enzymes of this class. Quite interestingly, we found that of the species that exhibit at least 50% enrichment on PS samples, 83% encode at least one long-chain alkane hydroxylase. This is significantly higher than in the random species we screened, and it implies that long-chain alkane hydroxylases have a greater significance towards PS degradability than AlkB and CYP153.

Furthermore, the majority of top PS-enriched species contained AlmA homologs of the family that exhibits the highest similarity to the AlmA identified from the species we used as query sequence, *A*. *baylyi*, and this species has been shown to emulsify and assimilate petroleum hydrocarbons [24]. Long-chain alkane hydroxylases are starting to be recognized as important contributors to the degradation of petroleum hydrocarbons [30, 32, 33], and there could be parallels between the species and enzymes that mediate biodegradation of petroleum and petroleum-based plastics.

### Enrichment of cyanobacteria and Deinococcus-Thermus on PS samples

At the phylum level, we found cyanobacteria to exhibit the strongest enrichment on PS compared to control samples. This was not due to repeat enrichment of a few cyanobacteria species in the majority of the PS samples we collected but rather the increased presence of a variety of cyanobacteria. This suggests that certain common properties of cyanobacteria grant these species growth advantages on PS. Cyanobacteria are autotrophs capable of fixing both inorganic carbon and nitrogen to organic compounds; in addition, they are known to produce a wide variety of compounds and tolerate extreme conditions. These special qualities have made cyanobacteria very valuable for agricultural and bioengineering purposes. In fact, multiple cyanobacterial strains have been reported to be able to degrade hydrocarbons [34–36], but we did not find common alkane hydroxylases (AlmA, LadA, AlkB, CYP153) to be present in the cyanobacteria species enriched on PS based on reference genome entries in NCBI.

Another interesting property of cyanobacteria is that most species produce C_15_-C_19_ alkanes for various uses related to cell membrane including maintaining cell shape and regulating cell division [37]. It would not be a stretch to imagine that cyanobacteria encode mechanisms to metabolize alkanes for homeostasis purposes, but there has not been reports of such. Some studies have rather focused on non-cyanobacteria strains with hydrocarbon degradative capabilities to play the role of hydrocarbon clearance in ecosystems [38], and this could also be an explanation for why we observed enrichment of alkane hydroxylases of non-cyanobacteria species on PS surfaces.

The phylum of Deinococcus-Thermus includes many species that are extremophiles or were isolated from hazardous environments. A few of the species have been explored in various types of bioremediation processes due to their abilities to tolerate harsh or toxic environments [39–41], and our finding that this phylum is enriched on PS waste samples imply that some of their species may be particularly capable of thriving on this surface, such as being able to use PS as a source of nutrient for growth.

Bacteria in the phyla of both Cyanobacteria and Deinococcus-Thermus are ones with unique and diverse metabolism that allows them to utilize or synthesize compounds and survive in unusual environments. This is possibly why these two clades were enriched on PS samples and warrants future research to look into the metabolic properties that enable them to colonize on PS.

## Supporting information

S1 Fig Alpha-diversity is lower in PS-associated microbiome than control soil samples. **A**, Simpson diversity indices of microbiomes associated with PS, S, and SN samples. **, P<0.05. **B**, Rarefaction curves for the PS, S, and SN samples.
(PNG)

S2 Fig Relative abundance of the most-detected species in the PS, S, and SN samples. The bar graphs on the left depict individual plastic samples of the three categories separately whereas the graph on the right exhibits combined results of the three sample types. Color codes of the different phyla are on the bottom.
(PNG)

S3 Fig Signature species from each of the PS, S, and SN samples as calculated using the LEfSe method to demonstrate that S- and SN-associated microbes are similar to each other while dissimilar to PS-associated species. (PNG)

S4 Fig Heatmap representation of metabolic pathway enrichment analyses based on the FAPROTAX database. **A**, Analysis for the Cyanobacteria and Deinococcus-Thermus species detected in our datasets. **B**, Functional enrichment analysis of PS-associated microbes without the Cyanobacteria and Deinococcus-Thermus species detected in our datasets. The relative abundance for each pathway is indicated individually for each sample.
(PNG)

S5 Fig Synteny analysis of predicted genes surrounding *almA* homologs in strains of *P*. *toyotomiensis* and *P*. *alcaliphila* whose whole genome sequences are available in the NCBI database. The blue, yellow, and green boxes represent forms I, II, and III of AlmA, respectively, as categorized in Fig 6A. Strain names are indicated on the left whereas gene names for AlmA homologs are indicated near the boxes.
(JPG)

S1 Table Location, pH, and vegetation of collection sites. (XLSX)

S2 Table Relative abundances of cyanobacteria and Deinococcus-Thermus species surveyed in the PS, S, and SN samples. (XLSX)

S3 Table Top 50 PS-enriched species ranked by average levels of PS-to-soil enrichment. (XLSX)

S4 Table Lists of top 30 PS-enriched species, random 30 species, and random 60 species used in our alkane hydroxylase analysis. (DOCX)
