# Supplementary figures and images for: Polystyrene-colonizing bacteria are enriched for long-chain alkane degradation pathways
Source: PLoS One. 2023 Oct 3;18(10):e0292137. doi: 10.1371/journal.pone.0292137 (PMC10547174; doi:10.1371/journal.pone.0292137)

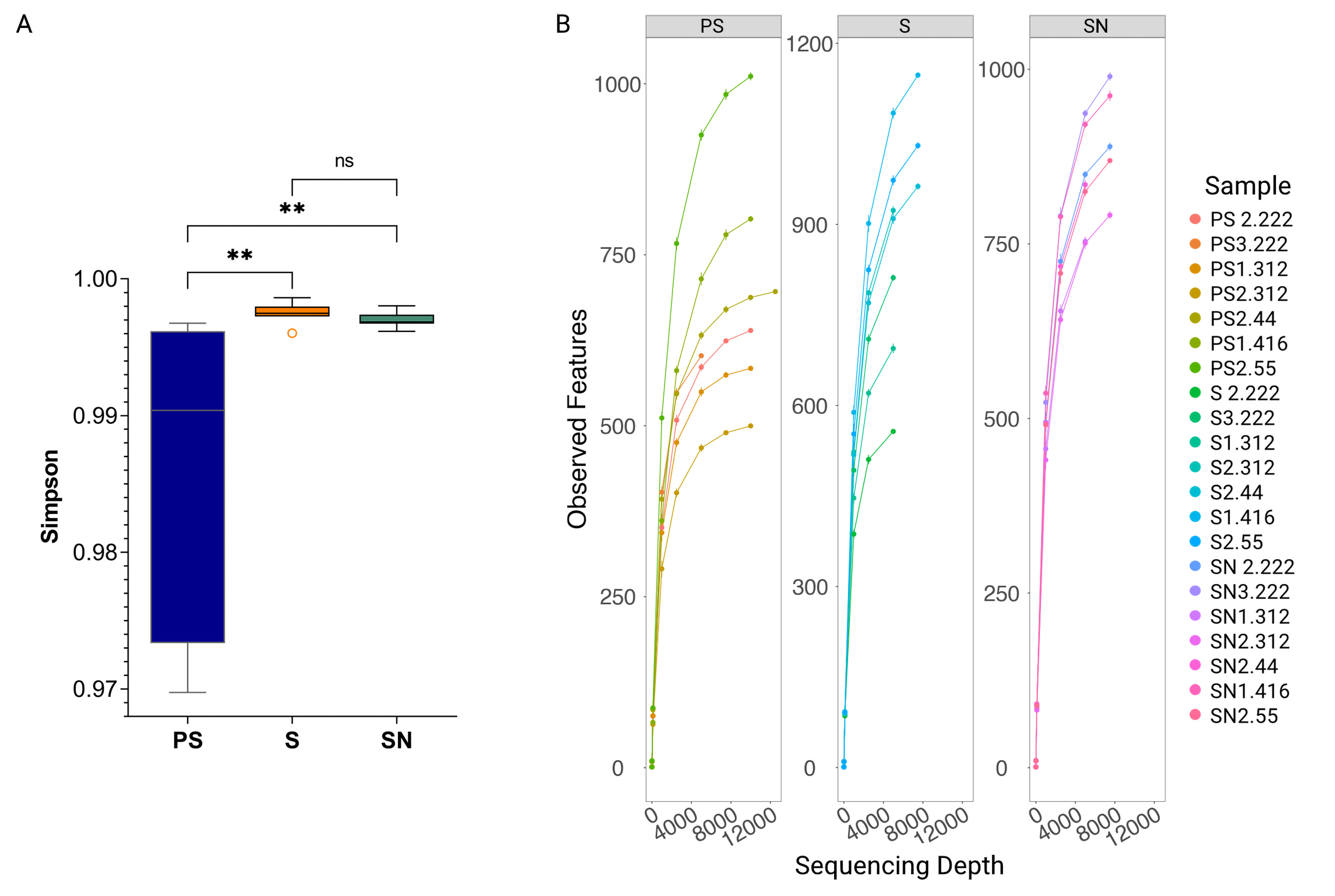

Supplement: S1 Fig — A, Simpson diversity indices of microbiomes associated with PS, S, and SN samples. **, P<0.05. B, Rarefaction curves for the PS, S, and SN samples. (PNG) [file pone.0292137.s001.png]

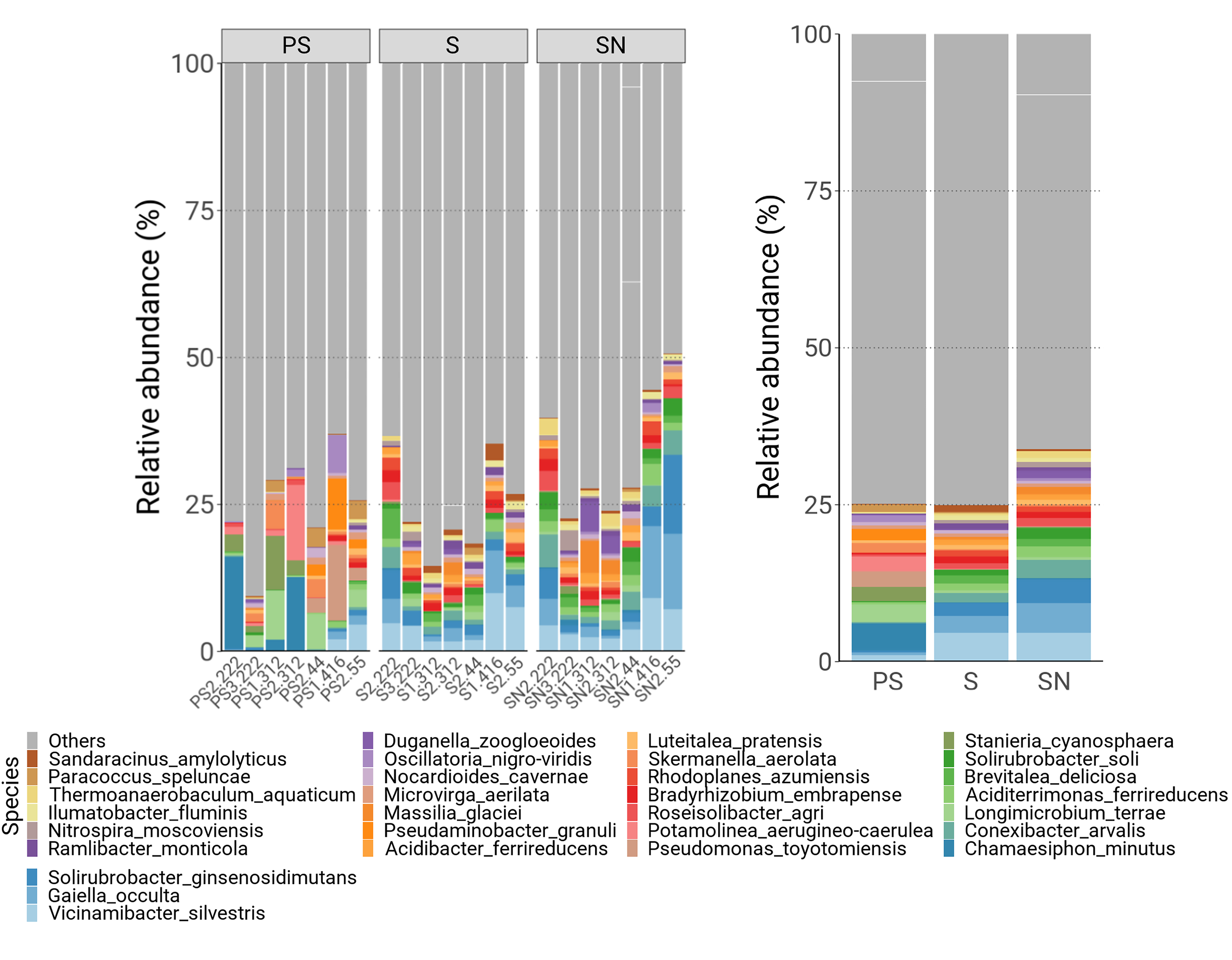

Supplement: S2 Fig — The bar graphs on the left depict individual plastic samples of the three categories separately whereas the graph on the right exhibits combined results of the three sample types. Color codes of the different phyla are on the bottom. (PNG) [file pone.0292137.s002.png]

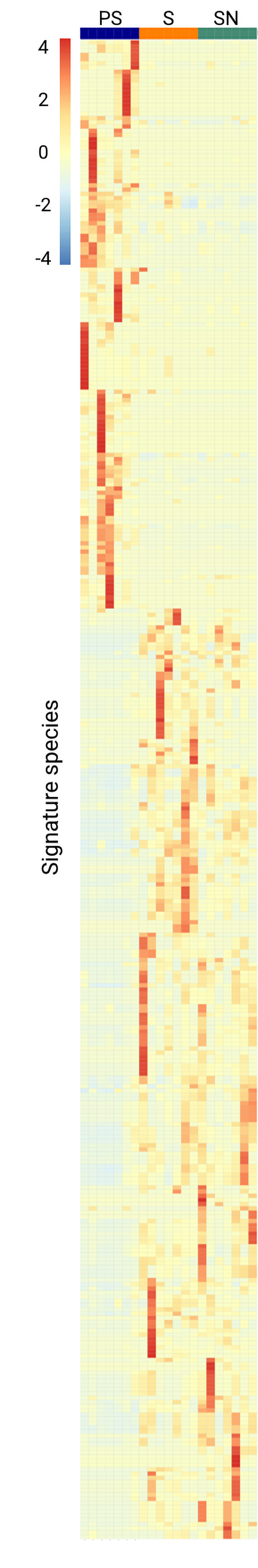

Supplement: S3 Fig — (PNG) [file pone.0292137.s003.png]

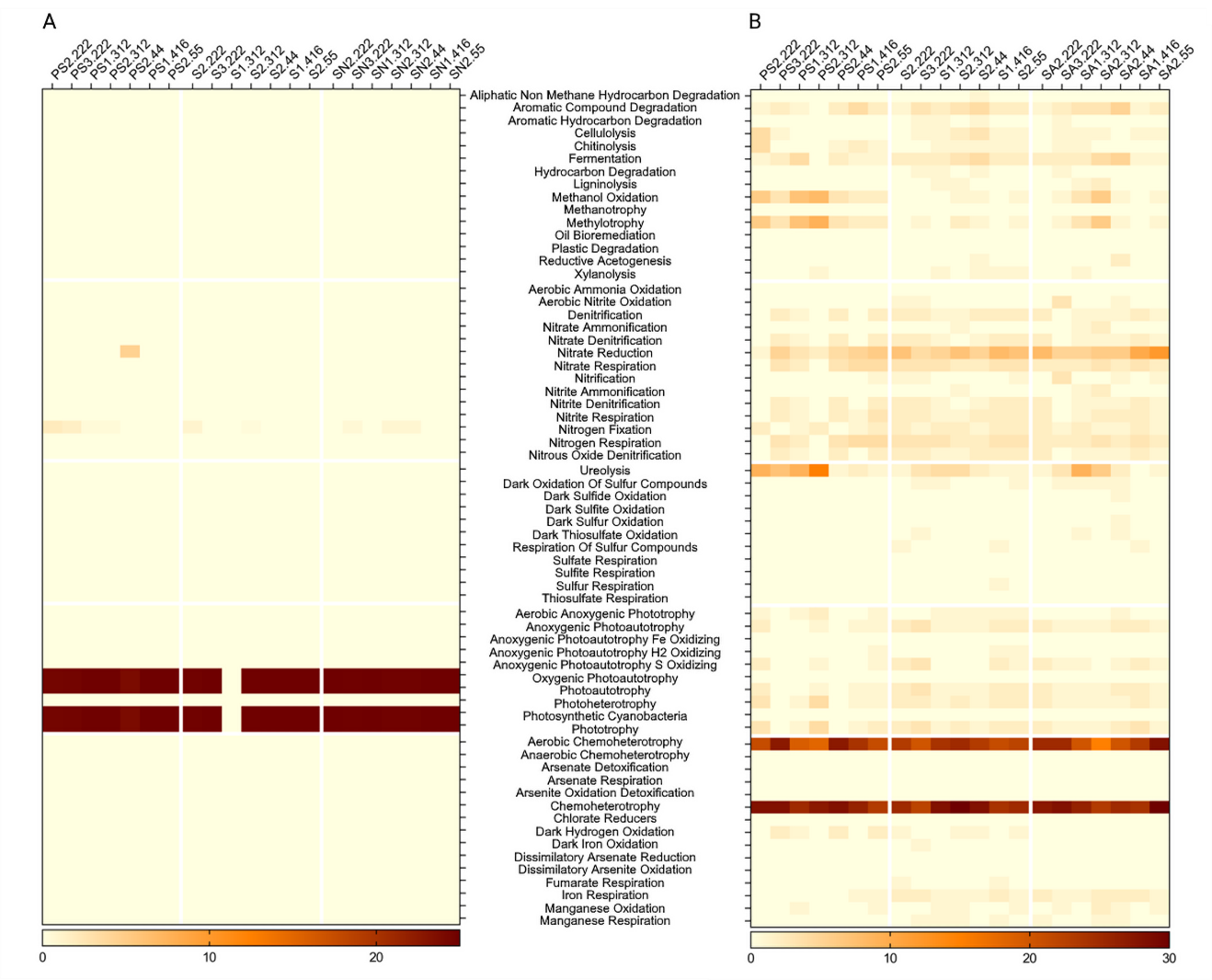

Supplement: S4 Fig — A, Analysis for the Cyanobacteria and Deinococcus-Thermus species detected in our datasets. B, Functional enrichment analysis of PS-associated microbes without the Cyanobacteria and Deinococcus-Thermus species detected in our datasets. The relative abundance for each pathway is indicated individually for each sample. (PNG) [file pone.0292137.s004.png]

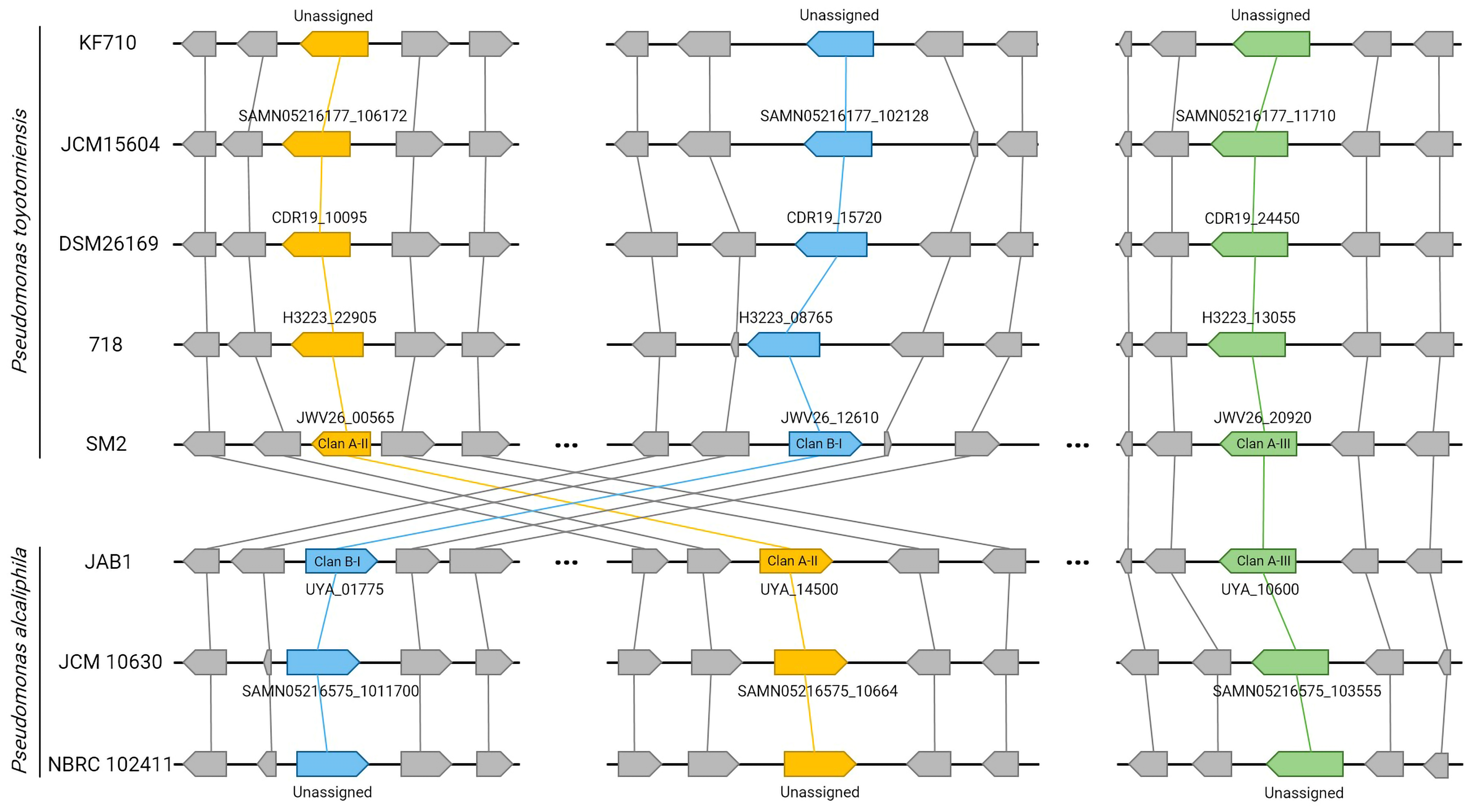

Supplement: S5 Fig — The blue, yellow, and green boxes represent forms I, II, and III of AlmA, respectively, as categorized in Fig 6A. Strain names are indicated on the left whereas gene names for AlmA homologs are indicated near the boxes. (JPG) [file pone.0292137.s005.jpg]
