# Supplementary material for: Polystyrene-colonizing bacteria are enriched for long-chain alkane degradation pathways
Source: PLoS One. 2023 Oct 3;18(10):e0292137. doi: 10.1371/journal.pone.0292137 (PMC10547174; doi:10.1371/journal.pone.0292137)
Supplement: S4 Table — (DOCX) [file pone.0292137.s009.docx]

**Table S4. Lists of top 30 PS-enriched species, random 30 species, and random 60 species used in our alkane hydroxylase analysis.**

| **Top 30 PS-enriched species** | **Random 30 species** | **Additional 30 random species*** |
| --- | --- | --- |
| *Pseudomonas toyotomiensis* | *Pseudorhodoplanes sinuspersici* | *Paracraurococcus ruber* |
| *Pseudomonas alcaliphila* | *Rhizobium halotolerans* | *Oceanibaculum pacificum* |
| *Crocosphaera watsonii* | *Planctopirus limnophila* | *Paracoccus litorisediminis* |
| *Methylobacterium oryzae* | *Skermanella rosea* | *Acidothermus cellulolyticus* |
| *Friedmanniella sagamiharensis* | *Flavobacterium anhuiense* | *Fluviicola chungangensis* |
| *Gloeobacter kilaueensis* | *Rhodoligotrophos appendicifer* | *Azonexus fungiphilus* |
| *Dankookia rubra* | *Patulibacter minatonensis* | *Fonticella tunisiensis* |
| *Sphingomonas ginsengisoli An et al. 2013* | *Mycobacterium asiaticum* | *Thermogutta terrifontis* |
| *Altererythrobacter rigui* | *Desulfomonile tiedjei* | *Clostridium moniliforme* |
| *Deinococcus humi* | *Methylobacterium durans* | *Bosea robiniae* |
| *Stenotrophomonas acidaminiphila* | *Flavobacterium hercynium* | *Brevibacterium casei* |
| *Methylobacterium platani* | *Mucilaginibacter pineti* | *Acinetobacter proteolyticus* |
| *Arthrospira platensis* | *Hyphomicrobium facile* | *Legionella dresdenensis* |
| *Bradyrhizobium elkanii* | *Inquilinus limosus* | *Legionella fallonii* |
| *Lapillicoccus jejuensis* | *Metabacillus litoralis* | *Raoultella planticola* |
| *Achromobacter insuavis* | *Ochrobactrum pseudogrignonense* | *Ideonella sakaiensis* |
| *Ochrobactrum lupini* | *Duganella sacchari* | *Luteitalea pratensis* |
| *Flavihumibacter profundi* | *Erythrobacter atlanticus* | *Rhodobacter ovatus* |
| *Blastococcus aggregatus* | *Saccharothrix espanaensis* | *Edaphocola flava* |
| *Sphingomonas melonis* | *Terrabacter tumescens* | *Ornithinicoccus hortensis* |
| *Amnibacterium kyonggiense* | *Paenarthrobacter nicotinovorans* | *[Empedobacter] haloabium* |
| *Paracoccus sediminis* | *Aminobacter aminovorans* | *Hyphomicrobium hollandicum* |
| *Deinococcus alpinitundrae* | *Sphingobacterium detergens* | *Aeromicrobium choanae* |
| *Spirosoma oryzae* | *Rhizobium daejeonense* | *Stella vacuolata* |
| *Sphingomonas hankookensis* | *Edaphocola flava* | *Geobacter argillaceus* |
| *Pseudokineococcus marinus* | *Nocardioides lianchengensis* | *Rhodococcus hoagii* |
| *Pseudonocardia endophytica* | *Litoreibacter ponti* | *Streptomyces brevispora* |
| *Moorea producens* | *Labedaea rhizosphaerae* | *Micropruina glycogenica* |
| *Adhaeribacter aerolatus* | *Clostridium combesii* | *Hyalangium minutum* |
| *Marmoricola scoriae* | *Mesorhizobium tianshanense* | *Moorella thermoacetica* |

*the two random 30 lists together make up the random 60 species list
